# Swim performance and thermoregulatory effects of wearing clothing in a simulated cold-water survival situation

**DOI:** 10.1007/s00421-015-3306-6

**Published:** 2016-01-29

**Authors:** Heather Bowes, Clare M. Eglin, Michael J. Tipton, Martin J. Barwood

**Affiliations:** Extreme Environments Laboratory, Department of Sport and Exercise Science, University of Portsmouth, Spinnaker Building, Cambridge Road, Portsmouth, PO1 2ER England, UK; Department of Sport, Exercise and Rehabilitation, University of Northumbria, Northumberland Road, Newcastle upon Tyne, NE1 8ST England, UK

**Keywords:** Thermoregulation, Swim failure, Cold water, Body composition, Hypothermia, Insulation

## Abstract

**Purpose:**

Accidental cold-water immersion (CWI) impairs swim performance, increases drowning risk and often occurs whilst clothed. The impact of clothing on thermoregulation and swim performance during CWI was explored with the view of making recommendations on whether swimming is viable for self-rescue; contrary to the traditional recommendations.

**Method:**

Ten unhabituated males (age 24 (4) years; height 1.80 (0.08) m; mass 78.50 (10.93) kg; body composition 14.8 (3.4) fat %) completed four separate CWIs in 12 °C water. They either rested clothed or naked (i.e. wearing a bathing costume) or swum self-paced clothed or naked for up to 1 h. Swim speed, distance covered, oxygen consumption and thermal responses (rectal temperature (*T*_re_), mean skin temperature (*T*_msk_) and mean body temperature *T*_b_) were measured.

**Results:**

When clothed, participants swum at a slower pace and for a significantly shorter distance (815 (482) m, 39 (19) min) compared to when naked (1264 (564) m, 52 (18) min), but had a similar oxygen consumption indicating clothing made them less efficient. Swimming accelerated the rate of *T*_msk_ and *T*_b_ cooling and wearing clothing partially attenuated this drop. The impairment to swimming performance caused by clothing was greater than the thermal benefit it provided; participants withdrew due to exhaustion before hypothermia developed.

**Conclusion:**

Swimming is a viable self-rescue method in 12 °C water, however, clothing impairs swimming capability. Self-rescue swimming could be considered before clinical hypothermia sets in for the majority of individuals. These suggestions must be tested for the wider population.

## Introduction

Accidental water immersion and consequent drowning is a worldwide problem resulting in an estimated 400,000–1,000,000 deaths each year (World Conference on Drowning Prevention 2011). Part of this statistic may be accounted for by a lack of basic swim capability and education on basic survival skills (Barwood et al. 2011). However, the disabling effects of low water temperature on swimming performance may also contribute to accidental death by drowning (Tipton 2003). When these statistics are combined with the estimate that up to  13.5 million adults take part in water-based leisure activities in the UK alone (Watersports Participation Survey 2013), it is evident that the responses to water immersion are of important research interest from the perspective of survival medicine and recreation.

Whether entering open water for leisure or by accident, if the water temperature is lower than that associated with the onset of vasoconstriction (i.e. 33 °C; Kenney et al. 2001) cardiovascular strain will be increased due to alterations in vasomotor tone and hydrostatic squeeze, thereby representing a critical temperature of water for inducing physiological strain (29–31 °C; Rennie et al. 1962). With lower water temperature, stimulation of peripheral cold thermoreceptors induces a cascade of responses described collectively as the Cold Shock Response (CSR; Tipton 1989). The response is characterised by an initial inspiratory gasp followed by uncontrollable hyperventilation, tachycardia and further vasoconstriction leading to an increase in blood pressure as a result of sympathetic nervous system activation (Tipton 1989). The loss of respiratory control increases the chance of aspirating water into the lung and death by drowning. At the peak of the CSR, swimming capability is significantly impaired. However, after the CSR subsides (3–5 min) it may be possible to swim a short distance to safety (Golden et al. 1986).

In cold water the decrement to exercise performance and risk posed to survival are not restricted to the initial minutes of immersion. Indeed, in the short term (i.e. up to 30 min) the onset of superficial muscle and nerve cooling results in an impaired capability to recruit motor units and generate force, thereby impairing muscle performance (Cheung et al. 2003; Davies and Young 1983; Ranatunga et al. 1987) and potentially swimming capability. Exercise increases muscle blood flow in accordance with metabolic demand and increases the rate of peripheral and central cooling by compromising the natural insulation provided by relatively unperfused resting muscle (Hayward et al. 1975a; Kenney et al. 1999; Toner et al. 1984; Veicsteinas et al. 1982).

If heat loss and muscle cooling cannot be compensated by increased insulation and metabolic heat production (shivering and/or exercise) then a significant deep body temperature drop will occur leading to the development of hypothermia (i.e. deep body temperature of ≤35 °C; Royal College of Physicians 1966; Sagawa et al. 1988). The point at which muscle performance declines to the extent that swimming can no longer be sustained and hypothermia ensues probably demarks the boundary between sports performance and survival.

The relationship between the thermal responses and physical performance is complicated by factors such as water temperature, activity level, body composition and external insulation in the form of clothing (Wallingford et al. 2000). Indeed, people are often clothed on entry to the water which increases buoyancy by trapping air within the clothing layers (Barwood et al. 2011). Subsequently this may promote the formation of a layer of still, relatively warmer water (“boundary layer”) at the skin which helps to reduce temperature gradients, thereby increasing insulation (Bullard and Rapp 1970). Consequently, clothing is of assistance on water entry and may play an important role in maintaining body heat if an individual chooses to remain stationary and await rescue, as is the historic recommendation (Hayward et al. 1975a, b; Keatinge 1961). However, it is unclear to what extent these properties would remain when movement takes place in the form of swimming in cold water.

It has been argued that attempted self-rescue by swimming could be a viable option in water temperatures as low as 10–14 °C despite the likely acceleration in muscle cooling rate (Ducharme and Lounsbury 2007). A number of studies in unclothed participants have demonstrated the ability of individuals to swim up to 1500 metres before becoming incapacitated by the cold (Knechtle et al. 2009; Tipton et al. 1999; Toner et al. 1984; Wallingford et al. 2000), in some cases for test durations of up to 90 min in 10 °C water (Tipton et al. 1999). It is possible, by way of providing insulation that clothing could be of further benefit to performance. However, the probable insulative benefit of wearing clothing whilst swimming must be balanced against the likely impairment to technique caused by an increase in drag resistance, and reduction in range of motion (Ohkuwa et al. 2002). It has already been shown in temperate water (29 °C) that outdoor, non-swim specific clothing reduces swimming velocity by 30.8 % and distance covered by 31 % compared to wearing a swimming costume (Ohkuwa et al. 2002). Moran (2014) also conducted tests in temperate water and not only reported similar decrements to speed (33 %) and endurance (28 %) in clothed participants (adolescents), but also reported that floatation capability was not influenced. However, the potential benefits or decrements to thermoregulation and performance have yet to be assessed in a cold-water survival scenario.

Accordingly, it was hypothesised that during cold-water immersion wearing clothing would significantly slow body cooling compared with being naked when both resting (*H*_1_), and swimming (*H*_2_); that swimming would significantly increase the rate of body cooling compared to resting (*H*_3_); and that clothing would significantly reduce swim distance through an increase in effort (*H*_4_).

## Methods

### Participants

The protocol received ethical approval from the Biosciences Research Ethics Committee. Ten healthy males (age 24 (4) years; height 1.80 (0.08) m; mass 78.50 (10.93) kg; body composition 14.8 (3.4) fat %; sum of skinfolds 36.8 (9.9) mm; O_2peak_ 3.0 (0.50 L min^−1^) provided written informed consent. They were all recreational swimmers, non-smokers, and with no recent repeated cold-water exposure.

### Experimental design

The study utilised a within-participant, repeated-measures design. Participants completed five tests in a swimming flume on separate days; one test of peak oxygen uptake (VO_2peak_) in 28 °C water on laboratory visit 1. The remaining 4 tests were conducted in 12 °C water and with participants being randomly assigned to a Latin Square design. These tests consisted of ‘swimming naked’ (i.e. bathing costume only), ‘swimming clothed’, ‘resting naked’ and ‘resting clothed’ conditions. Tests were separated by a minimum of 24-h, and completed at the same time of day (±1.5 h) within participant.

### Procedures

#### VO_2peak_

Following arrival at the laboratory, naked body mass and height were measured (in private) using calibrated weighing scales (Model I-10, Ohaus Corporation, New Jersey, USA) and a stadiometer, respectively (Leicester Height Measurer, Seca Ltd, UK); and were used to estimate body surface area using the equation of Dubois and Dubois (1916). After this, skinfold calliper measurements were taken at four sites; biceps, triceps, subscapular and suprailliac crest (Durnin and Womersley 1974) which were used to estimate body fat percentage using the Siri (1961) equation. Participants then changed into a pair of standard issue Lycra swimming shorts before being instrumented with a heart rate monitor (Team Polar, Finland). They then entered a 5.5 m (L) × 2.3 m (W) swimming flume (SwimEx Systems, USA) housed within an environmental chamber. The flume enabled water temperature control and reproducible increments in speed during all tests.

Prior to the VO_2peak_ test participants completed a standardised 5-min sub-maximal warm up. The flume speed was then set to 0.5 m s^−1^ following which the participant commenced breaststroke swimming in turbulent water. Breaststroke was selected for all tests as we contend that in a survival situation breaststroke would be adopted to enable navigation and keeping the airway clear of the water. Within the test the flume speed was increased every 2-min by approximately 0.15 m s^−1^ until the participant could no longer maintain the swim velocity or they reached volitional exhaustion. Heart rate was recorded at the end of each minute. At the end of the test participants momentarily breath-held whilst they self-inserted a mouthpiece and put on a noseclip before exhaling into a Douglas bag; they practised this procedure to mastery at rest prior to commencing the test. Three consecutive 20-s gas samples were taken to perform backward extrapolation calculations of peak oxygen consumption (Leger et al. 1980). Rating of perceived exertion (RPE; Borg 1982) was recorded at the end of the test. Swimming goggles were only permitted for the initial VO_2peak_ test.

#### Resting cold-water immersions

Participants self-inserted (in private) a calibrated rectal thermistor to measure rectal temperature (*T*_re_; Grant Instruments Ltd, Cambridge (Shepreth), UK) 15 cm past the anal sphincter and donned their Lycra swimming shorts. They were then instrumented with skin thermistors (Grant Instruments Ltd, Cambridge (Shepreth), UK) secured using adhesive tape (Tegaderm, 3M, Bracknell, UK) and tubed netting (Surgifix, BSN Medical GmbH, Hamburg, Germany) attached at four sites; bicep, chest, thigh and calf for subsequent calculation of mean skin temperature (*T*_msk_; Ramanathan 1964). *T*_re_ and *T*_msk_ data were subsequently combined to calculate mean body temperature for cold conditions (*T*_b_; Burton 1935). A heart rate monitor and a three-lead electrocardiogram (ECG) (LP15, Huntleigh Healthcare Ltd, Bedfordshire, UK) were also attached to the participants. They were then dressed in clothing (details below) if relevant to their test condition. Once dressed, participants entered the environmental chamber and sat in an immersion chair attached to a winch where baseline measures were taken and a 1-min expired gas sample was collected; having inserted a mouthpiece and donned a noseclip. The participant was then winched above the swimming flume for a further minute, before being lowered (rate of 8 m min^−1^) into the 12 °C still water to the level of the clavicle for 3-min. After 3-min participants removed their mouthpiece and noseclip, and remained as still as possible in the immersion chair for 1-h, or until core temperature reached 35.5 °C. A 1-min gas sample was collected every 15-min. Heart rate was recorded every 5-min, and skin and rectal temperatures were logged every 30-seconds (Squirrel 1000 series, Grant Instruments Ltd, Cambridge, UK). At the end of the test, participants exited the swimming flume and entered a hot bath (40 °C water) for rewarming.

#### Swimming immersions (12 °C)

Swimming immersions started with the same procedure as resting immersions. After the initial 3-min seated period participants removed the three-lead ECG and released themselves from the immersion chair. The flume was then brought up to a starting speed of ~0.5 m·s^−1^ and participants started to swim. From the initial starting speed participants were able to verbally prescribe their swimming pace by instruction to the researcher; they were instructed to find a pace that they could sustain if they had to swim for up to an hour in a survival situation. To enable the calculation of swimming speed and distance covered, continuous flume speed data were recorded by a portable flow metre (Streamflo 403, Nixon, Gloucestershire, UK) fixed in position for the duration of the tests connected to an analogue to digital converting system (PowerLab 16SP, ADI Instruments, Castle Hill, Australia) which recorded data every second at a resolution of 5 cm sec^−1^.

Expired air samples were taken every 15-min during the swim, along with reports of RPE (Borg 1982). After an hour of swimming, or once participants had reached a deep body temperature of 35.5 °C, the test was ended. The test was also terminated if the participants showed any consistent signs for impending swim failure or requested test cessation (i.e. volitional exhaustion). Swim failure was defined as the inability to keep the airway above the water line without standing, or the inability to remain swimming at the slowest possible flume speed. If testing ceased prematurely a final 1-min expired air sample was collected.

#### Clothing

In clothed conditions, participants wore jeans, t-shirt, woollen pullover, waterproof jacket, socks and canvas shoes. This clothing assembly corresponded to a ‘winter’ clothing assembly as stipulated in previous studies (Barwood et al. 2011). All clothing was of regular fit, was sized according to the participant’s build and provided by the experimenters.

#### Data analyses

Mean (SD) was calculated for thermal data at the point of termination of the immersion in each condition for subsequent comparison between conditions. To discern the effect of the clothing condition (i.e. clothed vs naked) and the activity type (resting vs swimming) on the rate at which hypothermia could develop, a rate of rectal temperature change was calculated for the minutes preceding the termination point of each immersion condition for each participant. This was based on visual evidence that a clear and linear cooling gradient had been established. Due to the influence of the experimental manipulations within the study, the number of data points available for analysis using this approach differed across conditions and participants. The average (SD) epoch available where a clear cooling gradient had been established in each condition was 26.0 (9.2), 23.8 (8.0), 22.4 (10.0) and 15.1 (9.7) min in the resting naked, resting clothed, swimming naked and swimming clothed conditions, respectively. These were converted to a cooling rate (°C h^−1^) using these time data and compared between conditions.

Data were checked for normality using a Kolmogorov–Smirnov test and were analysed using a repeated-measures ANOVA. A post hoc pairwise comparison was used to determine the direction of significant differences. Paired-samples *t* tests were undertaken to compare between clothed and naked swimming immersions for distance covered, average swim speed, VO_2peak_ and end RPE. Lastly, consistent with the procedures of Wallingford et al. (2000), Pearson’s correlation coefficients or Spearman’s rank order correlations were calculated (depending on data normality of distribution) between test duration and: body fat percentage; triceps, suprailliac crest skinfold thickness; swim speed; and rate of *T*_re_ cooling. For all statistical tests the *α*-level was set at 0.05.

## Results

### Swimming performance

From a total of 20 swimming tests, seven lasted the 60-min immersion time. In the other 13 tests, five were ended prematurely due to hypothermia, and the remaining eight because of unsustainable levels of perceived exertion. Unsustainable exertion levels were the predominant cause of withdrawal in the clothed condition, represented by a significantly higher end RPE value clothed [18 (1)] compared to when naked [16 (1); *t* = −2.75, *p* = 0.023).

Similar mean heart rates were evident whilst swimming clothed (139 [10] bpm) and naked (141 (16) bpm; *t* = *0*.134, *p* = 0.897). Volitional swim speed when naked was significantly faster (0.5 (0.1) m s^−1^) than when swimming clothed (0.4 (0.1) m s^−1^) (*t* = −2.41, *p* = 0.04). Yet oxygen consumption values at 15-min into the swim (the last common time point before the first participant dropped out) were not significantly different between naked (2.55 (0.37) L min^−1^) and clothed conditions (2.56 (0.35) L min^−1^) (*t* = −0.15, *p* = 0.89); inferring greater swimming efficiency in the naked condition. Participants in the naked condition swam for a significantly longer duration (52 (18) min) than when clothed (39 (19) min) (*t* = −2.27, *p* = 0.049). Consequently, a greater distance was covered when swimming naked (1264 (564) m than clothed (815 (482) m), (*t* = 3.13, *p* = 0.01).

### Resting immersions

In both resting conditions, all but two participants completed the 60-min immersion period. These two participants were withdrawn early due to low *T*_re_ values. For the resting conditions, similar average test durations were seen; clothed (60 (9) min), naked (57 (10) min) (*Z* = −1.07, *p* = 0.29). Yet there were significant differences in the heart rate evident at the last common time point of immersion before participant drop out (i.e. 30-min of immersion) (Naked HR 96 (19) bpm, clothed HR 82 (15) bpm; *t* = 2.496, *p* = 0.034) but oxygen consumption was variable and not significantly different (Naked 1.00 (0.33) L min^−1^, clothed 0.85 (0.18) L min^−1^; *t* = 1.738, *p* = 0.116) in the naked compared to clothed condition.

Irrespective of swimming or resting, when test exposure times were grouped into clothed or naked conditions, participants endured the immersions significantly longer when naked (56 (14) min), by 8-min compared, to when clothed (48 (18) min; *Z* = −2.34, *p* = 0.019). It is highly probable that these results reflect the early withdrawals in the clothed condition, due to the effort required to swim, as opposed to being indicative of any thermal benefits in either condition.

## Thermal responses

### Mean skin temperature (*T*_msk_)

**Fig. 1:**
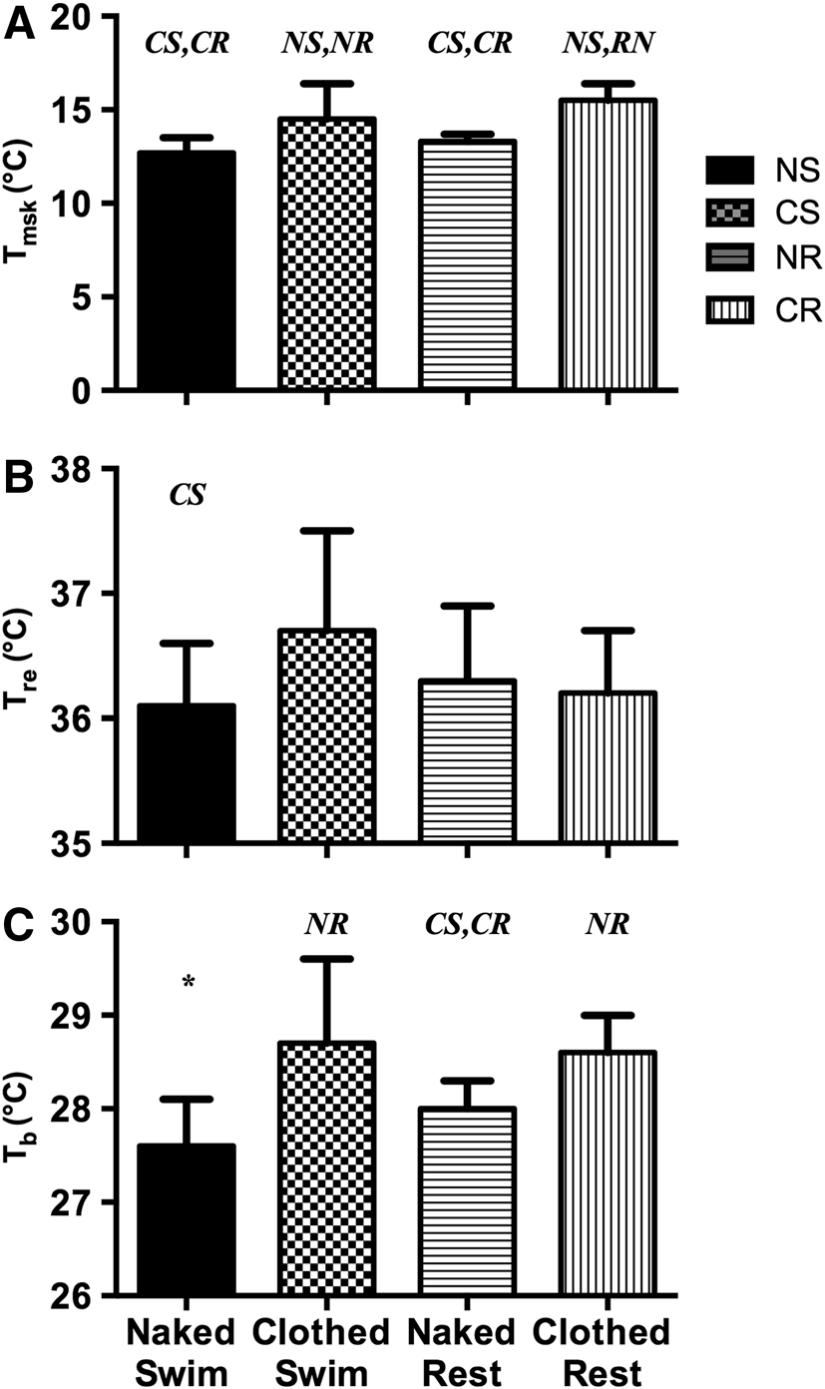
Mean (SD) *T*_msk_ (*panel A*), *T*_re_ (*panel B*) and *T*_b_ (*panel C*) at the point of test termination in the naked swim (NS), clothed swim (CS), naked rest (NR) and clothed rest (CR); *asterisk* indicates different to all conditions, italicised letters indicate which conditions are different to the bar they appear above (*n* = 10)

The experimental manipulations culminated in significantly different *T*_msk_ at the point of termination of immersion in each condition (*F*_(3,27)_ = 12.85, *p* = 0.001). Swimming naked produced the lowest *T*_msk_ at the end of the immersion which was significantly lower than swimming clothed (*p* = 0.015) and resting clothed (*p* = 0.001) but not different to resting naked (*p* = 0.083). Further differences in terminal *T*_msk_ were also evident between the swimming clothed and resting naked conditions (*p* = 0.043); being lower whilst resting naked. *T*_msk_ whilst swimming clothed was not different to the clothed condition at rest (*p* = 0.183). *T*_msk_ was also lower in the resting naked condition compared to the resting clothed condition (*p* = 0.001). These data are summarised in Fig. 1, panel A.

### Deep body temperature (*T*_re_)

The experimental manipulations culminated in significantly different *T*_re_ at the point of termination of immersion between two of the conditions (*F*_(3,27)_ = 3.55, *p* = 0.028). Swimming naked produced a significantly lower T_re_ at the end of the immersion compared to swimming clothed (*p* = 0.028); and was similar to the resting naked (*p* = 0.232) and resting clothed conditions (*p* = 0.528). Terminal rectal temperature was numerically highest in the swimming clothed condition, but this only approached being statistically significant compared to either resting naked (*p* = 0.067) or resting clothed (*p* = 0.060). The *T*_re_ seen at the end of the resting immersions was similar (*p* = 0.596), these data are summarised in Fig. 1, panel; B.

### Rates of *T*_re_ cooling

From a numerical perspective the rate cooling of T_re_, once a gradient for cooling had been established, was highest in the clothed swimming condition and tended to be highest when physical activity was undertaken [grouped mean for swimming −2.54 [1.9] °C h^−1^*cf* −2.11 (1.1) °C h^−1^ for resting]. However, no statistical differences were evident between conditions (*F*_(3,27)_ = 1.16, *p* = 0.342). These cooling rates were −2.45 (1.9) °C h^−1^, −2.63 (1.9) °C h^−1^ −2.15 (1.2) °C h^−1^, and −2.08 (1.1) °C h^−1^ in the swimming naked, swimming clothed, resting naked and resting clothed conditions, respectively. When the differences were calculated from the same time point after the CSR had subsided (i.e. after 3-min of immersion) the cooling rates were −1.97 (1.4) °C h^−1^, −1.56 (1.6) °C h^−1^ −1.44 (0.7) °C h^−1^ and −1.37 (0.8) °C h^−1^. Once again there were no differences between the conditions (*F*_(3,27)_ = 1.967, *p* = 0.143).

### Mean body temperature (*T*_b_)

The differences seen in the calculated *T*_b_ at the point of test termination was more closely reflective of the *T*_msk_ data and showed significant differences between conditions (*F*_(3,27)_ = 9.44, *p* = 0.001). Once again the lowest terminal *T*_b_ was seen in the swimming naked condition which was significantly lower than whilst swimming clothed (*p* = 0.007), and resting naked (*p* = 0.015) and clothed (*p* = 0.001). The highest estimated *T*_b_ was seen in the clothed swimming condition which was higher than resting naked (*p* = 0.018), but not different to resting clothed (*p* = 0.805). Terminal *T*_b_ was also higher in the resting clothed compared to resting naked condition (*p* = 0.004); see Fig. 1, panel C.

### Thermal correlation data

There was a significant relationship between Δ*T*_re_ and test duration for resting naked (*r* = 0.73) and clothed (*r* = 0.82), and when swimming naked (*r* = 0.75) (see Table 1). The reduced strength of the relationship when swimming clothed (*r* = 0.64) may be due to the early withdrawals as a result of the increased effort to swim when wearing clothes, suggesting that clothing may impair performance (i.e. precipitate early fatigue) more than they accelerate cooling for this condition.

**Table 1:** Correlations between physical characteristics, cooling rates and swim speed with test duration (*n* = 10)

	Clothed rest	Naked rest	Clothed swim	Naked swim
Mass (kg)	0.51	0.63*	0.53	0.42
Surface area (m^2^)	0.45	0.71*	0.63	0.41
SA: mass ratio (cm^2^ kg^−1^)	−0.56	−0.55	−0.32	−0.41
Body fat (%)	0.55	0.59	0.38	0.65
Triceps skinfold (mm)	0.43	0.59	0.60	0.75*
Biceps skinfold (mm)	0.10	0.27	0.57	0.29
Suprailliac crest skinfold (mm)	0.16	0.25	−0.10	0.05
Sum of skinfolds (mm)	0.55	0.59	0.36	0.65
Δ*T*_re_ (°C h^−1^)	0.82*	0.73*	0.64	0.75*
Swim speed (m s^−1^)	–	–	0.44	0.05

* Indicates significant correlation (*p* < 0.05)

### Body composition

Significant correlations with test duration were evident within the resting naked condition for body surface area (1.97 (0.16) m^2^; *r* = 0.71) and mass (*r* = 0.63) (see Table 1), but not SA:Mass ratio [253 (17) cm^2^ kg]. These relationships were not evident in the other conditions. These data suggest the test manipulations (exercise and clothing) present within the other three conditions confounded the relationship that was evident at rest. Whilst, no significant relationships were found between body fat percentage and test duration times, the data imply that body fat percentage is likely to have played a contributing role in determining test duration times particularly for the swimming (*r* = 0.65) and resting (*r* = 0.59) naked conditions (see Table 1). Despite this, no significant relationship was found between skinfold thickness at the suprailliac crest (10.7 (4.0) mm) and swimming duration (see Table 1). The only site specific skinfold with a significant correlation to test duration was at the triceps (8.6 (4.0) mm; *r* = 0.75; see Table 1) but not biceps (4.5 (2.2) mm).

## Discussion

This study examined the performance and thermoregulatory effects of wearing clothing compared to being naked in a simulated cold-water survival situation. The merits of awaiting rescue (resting immersions) and swimming to safety were explored with a view to providing recommendations that minimise homeostatic thermal disturbances or enable swim performance.

At rest it is evident that the presence of clothing maintained *T*_msk_ at a higher level than when naked; by 2 °C on average. When combined with deep body temperature data this culminated in a significantly higher mean body temperature despite similar test durations; therefore (*H*_1_) is accepted. When swimming, a similar picture emerged with *T*_msk_ maintained approximately 2 °C higher (on average) when wearing clothing compared to without (i.e. naked). This direction of difference also applied to our *T*_b_ data thereby supporting *H*_2_. In the case of the swimming tests, these results are complicated by the fact that test duration was longer in the naked swimming condition than when clothed, suggesting the reasons for test cessation were different between these two conditions. We suggest that hypothermia was more likely to precipitate test cessation in the naked swimming condition as this condition included the lowest terminal *T*_re_, whereas premature fatigue, rather than hypothermia is more likely when swimming whilst clothed. The fact that the thermal profiles were different at the end of the tests suggests that the boundaries of swim performance were not set by a common thermal threshold for absolute skin, deep body and mean body temperature. As a consequence, our data do not wholly support the hypothesis that cooling rate was higher when swimming compared resting and therefore *H*_3_ is only partially supported. Had this been the case we may have expected consistent thermal differences between naked conditions and clothed conditions whilst swimming compared to resting; *T*_msk_ and *T*_b_ tended to be numerically higher (grouped mean) at rest compared to whilst swimming by about 0.80 and 0.10 °C, respectively. There were no differences when rates of cooling were examined using *T*_re_ data. Likewise, the *T*_re_ data, which we consider to be the best index of the risk of hypothermia developing, only showed differences between clothed and naked swimming. However, our data do clearly support the suggestion that clothing would significantly reduce swim capability (*H*_4_) and the cause of this is a higher perceived exertion as the primary reason for test termination.

In the survival scenario, if the decision is taken to remain stationary in the water and await rescue, the higher level of surface insulation at the skin whilst clothed is achieved through the development of an insulating boundary layer of relatively warmer water that is retained between the clothing and the skin. Although this did not culminate in a higher *T*_re_ at the end of the experiment, the estimated *T*_msk_ suggested that the clothing resulted in some defence against convective heat loss, which would be heightened by a current of water passing over the skin. Consequently, in the clothed condition at rest, *T*_b_ was higher and heart rate was lower, implying a lower thermal strain, as a consequence of being clothed; although oxygen consumption was only numerically lower. We contend that the net effect over a longer duration of exposure than that used here would eventually be a slower decline in *T*_re_ and a lower physiological cost to thermoregulation (i.e. a lower oxygen consumption), due to less intense shivering in defence of deep body temperature. In the situation where clothing is not an impairment to remaining buoyant, retaining rather than removing clothing is sensible advice to slow the rate at which hypothermia develops. By contrast at rest whilst naked, thermal insulation is likely to be provided internally by relatively unperfused muscle in conjunction with body fat (Veicsteinas et al. 1982).

Upon the commencement of swimming, the internal insulation provided by unperfused muscle at rest is probably compromised. Veicsteinas et al. (1982) suggest that a modest exercise intensity, with a metabolic rate of in excess of 150 W m^−2^, is sufficient to compromise 80 % of the “variable” internal insulation provided by unperfused muscle; leaving only the “fixed” insulation of subcutaneous fat. Our tricep skinfold thickness data support the idea that the fixed insulation is also important in determining test duration whilst swimming. A higher skinfold in this area is likely to influence the rate of cooling of the arm muscles that are integral to propulsion whilst swimming which agrees with Wallingford et al. (2000) who offered a similar finding when studying front crawl swimming. In our study the average (SD) metabolic rate in the swimming conditions was 2.56 (0.36) L min^−1^; well in excess of the threshold identified by Veicsteinas et al. (1982) hence we would expect the variable insulation evident at rest to be largely compromised thereby isolating the fixed insulation as a key factor. We also showed that body surface area and to a lesser extent body fat could influence test duration although in the latter case the statistical evidence of this relationship was weak. Consequently, hypothermia may result in lean individuals with a large surface area for heat exchange if the safe refuge of land and shelter is not achieved within a reasonable swimming distance. This will be sooner if clothing, and a resultant insulating boundary layer of water, is absent.

We also show that swimming performance was better in the absence of clothing. The direction of these differences agree with observations made in temperate water, but our loss in speed (20 %) and endurance (36 %) was lesser and greater, respectively, than that noted by Ohkuwa et al. (2002) and Moran (2014) in more temperate conditions. This could be due to a more conservative speed being possible in cold water, our testing of a different cohort of participants by comparison to others and, in the case of the loss in endurance, the greater debilitating effect of the cold. Indeed, specific to cold water performance our data show that it is possible to swim an average of 1264 m in 12 °C water when naked, compared to a considerably shorter distance of 815 m when clothed; these distances were achieved over an average test duration of 52- and 39-min, respectively. When considered in relation to swimming pace, a rate of 27.2 m min^−1^ was evident when naked which was reduced to 23.4 m min^−1^ when clothed. These performance data lie within the boundaries identified by Ducharme and Lounsbury (2007), although these authors did not distinguish the consequent impact of clothing; this represents a novel aspect to our study. Taking the findings presented above together: before attempting to swim to safety in cold water the decision to remove clothing to improve swimming efficiency and delay fatigue must be balanced against the increased risk of developing hypothermia if in the water too long. The logistics of removing the clothing whilst immersed, prior to swimming, may also be problematic; we did not test this capability in the present study. Collectively, it would appear that, under the conditions of the present tests, if safety is within an approximate range of 1300 m or can be reached within 50 min, swimming is more likely to be successful in the absence of clothing; which appears to impose more of a performance burden than a thermoregulatory advantage.

Our study is not without limitation. Our study clearly applies to the situation where continuous swimming is undertaken to achieve survival. Our protocol did not allow for recovery stops which may have produced different results. The data analyses of the swimming conditions compared different test durations because the reason for test termination was different between the naked (i.e. thermal reasons) and clothed swimming conditions (i.e. fatigue). It was important that these tests were of self-paced swimming and conducted to swim failure as would be the case in the survival scenario; hence we uncovered differences in participant attrition. An alternative is to look at a fixed intensity protocol or examine the last common time point before participants began to be withdrawn from the study for reasons of safety (i.e. impending hypothermia) or exhaustion; we had to adopt this latter approach in the case of some variables we measured (e.g. VO_2_). This would have restricted our analysis to a 15-min epoch across the cohort which was not sufficiently long enough to establish differences between tests in some of our variables. We attempted to account for this limitation in part by additionally calculating rates of cooling (°C h^−1^). However, this might not be long enough to establish a gradient for cooling, and thus long enough to realistically discern the possible increased risk of hypothermia. Hayward et al. (1975b) considered the lower limit of body temperature to maintain ‘useful activity’ was 33 °C. Extrapolation of our deep body temperature cooling data, assuming a start point of 37.5 °C, would suggest that 33 °C would be reached as a consequence of swimming (irrespective of clothing level), 41-min quicker than when resting and by 22-min faster when naked compared to clothed (irrespective of activity level). Clearly our cooling data apply only to thermal changes in 12 °C water in a population with relatively low body fat and may vary accordingly with increases in body fat mass or distribution (McArdle et al. 1984a, b), differences in gender and age (Cannon and Keatinge 1960; Tarlochan and Ramesh 2005; Tipton et al. 1998; Xu et al. 2007) as well as different temperature water (Tipton et al. 1998). Likewise, our cooling data only represent an estimate based on a two-compartment thermometry model of weighted deep body and skin temperature from four sites. Ideally more skin thermistor sites would have been used to capture the probable non-uniform temperature distribution that is likely to prevail in cold conditions (Toner et al. 1984); enabling unrestricted swimming movements was our reason for our methodological choice. Further research is clearly warranted to enable generalisation from the present survival scenario to a wider population and set of circumstances. However, collectively our analyses have enabled our experimental hypotheses to be interrogated which we feel justifies our approach.

In summary, the clothing assembly attenuated mean skin and mean body temperature decline during immersion in 12 °C water. At rest, retaining clothing is clearly of thermal benefit and would probably remain so if long-term static immersion occurred. Wearing clothing when swimming played a significant role in promoting the early onset of swimming failure because of increased physiological cost to swimming movements making the swimming stroke less efficient. The thermal benefit of retaining clothing was outweighed by the negative effect of clothing on swim performance and, if safe refuge (i.e. land) is within a maximum of 1300 m swimming distance, clothing could be removed to enable an individual’s optimum swimming performance. However, an overestimation of swim capability or underestimation of the distance to safe refuge in a life-threatening situation may lead to the early onset of hypothermia and death by drowning.

## Abbreviations

CR: Clothed rest
CS: Clothed swim
CSR: Cold shock response
CWI: Cold-water immersion
HR: Heart rate
NR: Naked rest
NS: Naked swim
RPE: Rating of perceived exertion
T_b_: Mean body temperature
T_msk_: Mean skin temperature
T_re_: Rectal temperature
O_2peak_: Peak oxygen consumption
